# Diabetic microenvironment preconditioning of adipose tissue-derived mesenchymal stem cells enhances their anti-diabetic, anti-long-term complications, and anti-inflammatory effects in type 2 diabetic rats

**DOI:** 10.1186/s13287-022-03114-5

**Published:** 2022-08-19

**Authors:** Wanlu Su, Songyan Yu, Yaqi Yin, Bing Li, Jing Xue, Jie Wang, Yulin Gu, Haixia Zhang, Zhaohui Lyu, Yiming Mu, Yu Cheng

**Affiliations:** 1 School of Medicine, Nankai University, No. 94 Weijin Road, Tianjin, 300071 China; 2 Department of Endocrinology, Chinese People’s Liberation Army General Hospital, No. 28 Fuxing Road, Beijing, 100853 China; 3 Department of Endocrinology, Beijing Tiantan Hospital, Capital Medical University, Beijing, 100070 China; 4 Department of Endocrinology, Diabetes Center of People’s Liberation Army (PLA), PLA Strategic Support Force Characteristic Medical Center (The 306th Hospital of PLA), Beijing, China; 5 Department of Endocrinology, Beijing Tiantan Hospital, Capital Medical University, Beijing, 100070 China; 6 Beijing Friendship Hospital, Capital Medical University, Beijing, China

**Keywords:** Diabetes, Diabetes complications, Mesenchymal stem cells, Diabetic microenvironment precondition

## Abstract

**Background:**

Mesenchymal stem cells (MSCs) exert anti-diabetic effects and improve long-term complications via secretory effects that regulate macrophage polarisation and attenuate inflammation. Enhancing the efficacy of MSCs needs to be explored further. The in vitro culture microenvironment influences the secretory profile of MSCs. Therefore, we hypothesised that a diabetic microenvironment would promote the secretion of cytokines responsible for macrophage polarisation, further attenuating systemic inflammation and enhancing the effects of MSCs on type 2 diabetes (T2D) and long-term diabetic complications.

**Methods:**

Preconditioned adipose-derived mesenchymal stem cells (pre-ADSCs) were obtained after co-cultivating ADSCs in a diabetic metabolic environment (including high sugar, advanced glycation end-product, and lipopolysaccharides). The regulatory effects of pre-ADSCs on macrophages were observed in vitro. A T2D rat model was induced with a high-fat diet for 32 weeks combined with an intraperitoneal injection of streptozotocin. Sprague–Dawley (SD) rats were divided into four groups: normal group, diabetes without treatment group (PBS), ADSC treatment group, and pre-ADSC treatment group. ADSCs and pre-ADSCs were intravenously administered weekly to SD rats for 6 months, and then glucose homeostasis and long-term diabetic complications were evaluated in each group.

**Results:**

The secretion of cytokines related to M2 macrophage polarisation (IL-6, MCP-1, etc.) was increased in the pre-ADSC group in the in vitro model. Pre-ADSC treatment significantly maintained blood glucose homeostasis, reduced insulin resistance, promoted islet regeneration, and ameliorated the complications related to diabetes in rats (chronic kidney disease, non-alcoholic steatohepatitis, lung fibrosis, and cataract) compared to the ADSC group (*P* < 0.05). Additionally, the number of anti-inflammatory M2 macrophage phenotypes was enhanced in tissues following pre-ADSC injections. Moreover, the expression of pro-inflammatory genes (iNOS, TNF-α, IL-1β) was reduced whereas that of anti-inflammatory genes (Arg1, CD206, and Il-10) was increased after cultivation with pre-ADSCs.

**Conclusion:**

Diabetic microenvironment-preconditioned ADSCs effectively strengthen the capacity against inflammation and modulate the progress of long-term T2D complications.

**Supplementary Information:**

The online version contains supplementary material available at 10.1186/s13287-022-03114-5.

## Background

Type 2 diabetes (T2D) is a metabolic disorder that is closely associated with the long-term dysfunction of various organs, which is the main cause of death or disability in T2D. T2D is related to serious damage to blood vessels, resulting in non-alcoholic steatohepatitis, chronic kidney disease (CKD), cataract, and fibrosis of the lung caused by poor control of blood glucose [1]. Diabetic nephropathy (DN) is a major cause of end-stage renal failure worldwide, and patients with end-stage DN have a poor prognosis [2]. Currently, traditional anti-diabetic drugs provide only palliative relief for diabetic complications, and there is no definitive cure. Therefore, new therapeutic strategies that reverse these clinical outcomes are urgently needed.

Chronic low-level inflammation has recently been regarded as a factor related to diabetic complications [3]. Diabetes is accompanied by the elevated release of local and systemic inflammatory factors which play a paracrine role resulting in pancreatic islet dysfunction and insulin resistance in tissues, that may promote the occurrence and development of T2D complications [4, 5]. Moreover, excessive activation of inflammatory pathways in macrovascular (coronary artery disease, peripheral artery disease, and stroke) and microvascular (nephropathy, neuropathy, and retinopathy) complications has been confirmed to exist in patients with T2D [6–8]. Macrophages are the dominant immune cells that lead to enhanced inflammation in T2D [9]. According to their functions, activated macrophage have been broadly divided into two categories: classically activated macrophages (M1 type) and alternatively activated macrophages (M2 type). The proportion of mutual transformation between M1 and M2 governs the organ’s fate in the deregulated inflammatory response, which has been widely demonstrated in previous studies [10, 11]. A review showed that enhanced inflammatory signals produced by M1 macrophages and attenuated anti-inflammatory cytokines produced by M2 could lead to persistent fibrosis of DN.

Mesenchymal stem cells (MSCs) are adult stem cells that have the potential for multidirectional differentiation and self-replication [12]. In recent years, the immunomodulatory function of MSCs has become increasingly important. Importantly, it has been found that MSCs possess properties of alleviating chronic inflammation in tissues and protecting organ functions by regulating macrophage polarisation in a variety of disease models [13, 14]. Consistently, our previous studies showed that umbilical cord-derived MSCs elicit an anti-inflammatory phenotype by secreting IL-6 and MCP-1 to alleviate insulin resistance and promote islet function in an early onset diabetic model. In addition, we established a rat model to closely mimic the long-term complications occurring in T2D and showed that multiple intravenous infusions of adipose-derived mesenchymal stem cells (ADSCs) attenuated systemic inflammation, altered the tissue M1/M2 ratio, partly promoted glucose homeostasis, and alleviated long-term diabetes complications, such as lung, liver, kidney, and cardiovascular complications [15]. The aim of this study was to enhance the effects of MSCs on glucose homeostasis and long-term diabetic complications.

Accumulating evidence shows that the tissue microenvironment can influence the secreted soluble factors by MSCs [16, 17]. Previous studies have confirmed that inflammation irritation and appropriate low-oxygen culture conditions enhance the secretory effects of MSCs [18, 19]. Based on the findings of previous studies, MSCs appear to possess a ‘short-term memory’ ability to secrete a large number of related cytokines when re-exposed to the similar environmental stimulation or danger signals [20]. Therefore, we hypothesised that pretreatment with a diabetic metabolic environment, including high sugar, advanced glycation end-product (AGE), free fatty acids, and lipopolysaccharides (LPS), would promote the secretion of cytokines responsible for macrophage polarisation, further attenuate systemic inflammation, and enhance the effects of MSCs on long-term diabetes complications.

In this study, we showed that ADSCs preconditioned in a diabetic microenvironment exert superior effects on glucose homeostasis and long-term T2D complications than normally cultured MSCs. Moreover, infusions of preconditioned ADSCs further enhanced the M2/M1 ratio in multiple organs and attenuated systemic inflammation, possibly via increased secretion of IL-6 and MCP-1.

## Methods

### Isolation and identification of ADSCs

ADSCs were isolated, cultured and identified as described by Yu et al. [4]. Briefly, ADSCs were collected from the subcutaneous adipose tissue of male Sprague–Dawley (SD) rats (weighing 60–80 g). The cells were resuspended in low-glucose Dulbecco’s modified Eagle’s medium (DMEM; Gibco, USA) containing 10% foetal bovine serum (FBS; Gibco) and 1% penicillin–streptomycin (Gibco). The cultures were maintained at 37 °C with 5% CO_2_/95% humidity. The culture medium was replaced after 24 h to remove non-adherent cells, and the new culture was replaced twice a week. The immune phenotype of the cultured ADSCs was characterised using flow cytometry at passage 3.

### Diabetic microenvironment preconditioning of ADSCs

ADSCs at passage 3 were grown in 6-well plates (3 × 10^4^/well). After overnight culture, the diabetic microenvironment pretreatment group was co-cultured in two different concentrations of stimuli (low concentration precondition: LPS (Abcam, MA) 1 µg/mL, AGE (Abcam) 1 µg/mL, and glucose (Proteintech, Wuhan, China) 2.5 mg/ml; high concentration precondition: LPS 2 µg/mL, AGE 5 µg/mL, and glucose 4.5 mg/mL). After incubating for 24 h, 48 h, and 72 h, the medium was changed to low-sugar DMEM containing 10% FBS without stimulation, as in the normal control group.

### Extraction, culture, and induction of rat peritoneal macrophages

Male SD rats weighing 110 ± 10 g were sacrificed by cervical dislocation or by overdose of 3% pentobarbital injected in the tail vein. The rat was soaked in 75% alcohol for 10 min and then placed on a clean bench. Following fixing of the rat, the abdominal skin and muscle were separated with sterile scissors and tweezers, and the abdominal muscle was wiped three times with 75% alcohol. The abdominal wall was lightly lifted with a pair of tweezers and a 10 mL syringe was used to inject 10 mL of serum-free high-sugar DMEM. Cotton balls soaked in 75% alcohol were used to continuously massage the abdominal wall to ensure that the culture medium made full contact with the abdomen. After 7 min, a syringe was used to suck up the liquid in the abdomen. The pink solution turned pale yellow. The liquid was transferred to a 15 mL centrifuge tube and centrifuged at 1000 rpm for 5 min. The supernatant was discarded, and the cells were suspended in high-glucose DMEM containing 10% FBS. The cells were then inoculated into a 6-well plate. Cells from one rat were used to inoculate six wells. The cells were placed in a 5% CO_2_ incubator at 37 °C overnight, followed by gentle rinsing with phosphate buffered saline (PBS, Proteintech) solution three times the next day to remove red blood cells and other non-adherent cells. At this time, M0 type macrophages were obtained. The induction method of pro-inflammatory macrophages involved addition of LPS to 10% FBS high-sugar DMEM the next day when the medium was changed. The final concentration was 1 µg/mL. After stimulation for 24 h, the cells were gently washed three times with PBS. At this time, M1 type macrophages were obtained.

### Identification of rat peritoneal macrophages

The extracted macrophages were seeded in a 6-well plate containing sterile glass slides in advance, incubated in a 37 °C incubator with 5% CO_2_ overnight, and then gently rinsed with PBS solution three times to remove red blood cells. After incubation for 48 h, the cells were washed three times with PBS. After fixing with 4% paraformaldehyde for 15 min, the cells were washed with PBS three times for 5 min each time. The cells were then treated with 0.3% Triton for 5 min, the supernatant was discarded, and washed three times with PBS for 5 min each time. An immunohistochemical pen was used to circle the stained area on the slides. Non-specific sites were blocked with goat blocking serum for 30 min. The slides were incubated with anti-F4/80 antibody (Abcam, 1:250) overnight at 4 °C. On the second day, the cells were washed three times with PBS solution for 7 min each time, and incubated with an anti-mouse A594 fluorescent secondary antibody (Abcam, 1:500) for 2 h. The cells were washed three times with PBS solution for 7 min each time. Nuclei were stained with 4′,6-diamidino-2-phenylindole (DAPI, Sigma-Aldrich, USA, 1:2000) for 5 min. The cells were observed under a laser confocal scanning microscope and images were captured.

### Co-culture of ADSCs and macrophages

On the first night, the third-generation ADSCs were seeded in the transwell cells at 1.5 × 10^4^ cells/well. The next morning, the pre-ADSC group was treated with LPS, AGE, and glucose for 24 h. The induced M1 macrophage chamber was aspirated to remove the supernatant, washed three times with PBS solution, and the ADSC and pre-ADSC chambers were placed on top of the M1 macrophages. After 72 h of co-cultivation, the macrophages were used to extract proteins, RNA, or for immunofluorescence experiments.

### Detection of cell apoptosis and cell proliferation

ADSCs (Passage 3, 1 × 10^3^ cells/well) were seeded in ten wells of a standard 96-well plate with 100 µL low-sugar DMEM containing 10% FBS, and an equal amount of PBS solution was added to the remaining wells. After incubating overnight, the media in five out of ten wells were replaced with the diabetic microenvironment pretreatment medium, while that in the other five wells were replaced with the ordinary low-sugar DMEM containing 10% FBS. After incubating for 24 h, 20 µL methylthiazolyldiphenyl-tetrazolium bromide (MTT, Elabscience, Wuhan, China) solution (5 mg/mL, 0.5% MTT) was added to each well and incubated further for 4 h. The supernatant was aspirated, and the blue-purple precipitate at the bottom of the cell was saved. A total of 150 µL dimethyl sulfoxide (DMSO, Elabscience) was added to each well and the plate was shaken on a shaker at a low speed for approximately 10 min to fully dissolve the crystals. The absorbance of each well was measured at 490 nm using an enzyme-linked immunoassay (Thermo Scientific, CA, USA).

### Animal experiment, treatment, and tissue sampling

SD rats (8-week-old, male) were fed for 8 weeks with a high-fat diet (HFD; 60% fat, Research Diets, New Brunswick, NJ) or a normal chow diet (NCD). The HFD-fed rats were intraperitoneally injected with a single dose of 25 mg/kg streptozotocin (STZ, Sigma-Aldrich, St. Louis, MO, USA) in 10 mmol/L citrate buffer (pH 4.5). All STZ-treated rats with random glucose levels higher than 16.7 mmol/L were considered to be T2D rats. The T2D rats were fed a HFD (6 months) to form a long-term T2D complication rat model. At 6 months following the STZ injection, the T2D rats were randomly divided into three groups: normal ADSC treatment group (ADSCs: rats were weekly infused through the tail vein with 3 × 10^6^ ADSCs suspended in 0.5 mL PBS); diabetic untreated group (T2D: rats received the same amount of PBS weekly via tail vein injection); diabetic microenvironment-preconditioned ADSC treatment group (pre-ADSCs: rats were weekly administered 3 × 10^6^ pre-ADSCs suspended in 0.5 mL PBS through the tail vein), and NCD-fed rats (Normal) served as the control group. After infusing for 6 months, intraperitoneal glucose tolerance test (IPGTT), intraperitoneal insulin tolerance test (IPITT), and hyperinsulinaemic-euglycemic clamp experiments were performed. Fasting insulin (FINS) levels and glucose infusion rate (GIR) were monitored. Homeostatic model assessment of insulin resistance (HOMA-IR) = fasting blood glucose (FPG, mmol/L) × FINS (mIU/L)/22.5. For IPGTT/IPITT, rats were fasted overnight and then intraperitoneally injected with 50% sterile glucose solution at a dose of 2 g/kg or insulin at a dose of 1 U/kg. Blood glucose levels were measured at 0, 30, 60, 90, and 120 min after injection. Whole blood was collected from the left ventricle, and plasma was obtained after centrifugation at 3500 rpm for 20 min. The serum concentrations of insulin were measured using an enzyme-linked immunosorbent assay (ELISA) Kit (R&D Systems, Minneapolis, MN, USA). Blood lipids (including low-density lipoprotein cholesterol [LDL-C], total cholesterol, and triglyceride [TG]) and hepatic enzymes (alanine aminotransferase [ALT] and aspartate aminotransferase [AST]) were measured by the Servicebio Corporation (Wuhan, China). Rats were sacrificed and all tissues (including spleen, liver, kidney, heart, lung, pancreas, etc.) were harvested as described in our previous study [17]. All experimental procedures were approved by the Medical Ethics Committee of the Chinese PLA General Hospital.

### Haematoxylin and eosin (H&E) staining, immunohistochemistry, and immunofluorescence

The kidney, liver, lung, and heart were embedded in paraffin as per standard protocol, sectioned into 4–6 μm thin sections, and then the tissues were stained with H&E, periodic acid-Schiff (PAS), Masson’s trichrome, Sirius Red, or immunohistochemically stained with primary antibodies against collagen type I (1:500, rat, Abcam) and α-smooth muscle actin (1:1000, rat, Abcam). A light microscope (Olympus, Japan) was used to observe the morphological structure of each tissue for histological analysis as described in our previous study [4].

For immunofluorescence analysis, the frozen tissues were cut into 6 μm sections and incubated for 14 h at 4 °C with primary antibodies against insulin (1:200, guinea pig, Abcam), glucagon (1:2000, mouse, Abcam), F4/80 (1:100, rat, Santa Cruz), iNOS (1:100, rat, Abcam), Arg (1:100, rabbit, Abcam), collagen type I (1:500, rabbit, Abcam), and α-smooth muscle actin (1: 100, mouse, Sigma-Aldrich), followed by incubation with Alexa Fluor 488/594-conjugated secondary antibodies (1:500, Invitrogen, USA) at room temperature for 2 h. The nuclei were stained with DAPI. The immunofluorescently stained sections and cells were examined and photographed using a laser scanning confocal microscope (Leica, Wetzlar, Germany).

### Quantitative real-time reverse transcriptase polymerase chain reaction (qRT-PCR)

Total RNA was extracted from macrophages grown in the different co-culture systems using the TRIzol reagent (Invitrogen, Thermo Fisher Scientific, USA) and reverse-transcribed to single-stranded cDNA using a reverse transcription kit (Thermo Scientific) following the manufacturer’s instructions. qRT-PCR analysis was performed using a SYBR® Green PCR Master Mix (Applied Biosystems) on an ABI Prism thermal cycler (Applied Biosystems, CA, USA). The thermal cycling programme involved incubation at 50 °C for 2 min, 94 °C for 5 min, followed by 94 °C for 30 s, 60 °C for 30 s, and 72 °C for 30 s for 40 cycles. Melting curve analysis was performed to ensure primer specificity. All gene expression levels were normalised to the GAPDH levels. The primers used are listed in Additional file 1: Supplementary Table 1.

### Western blotting

Western blot analysis was performed as described previously [7]. The antibodies used included Arg-1 (1:1000, Abcam), iNOS (1:1000, Cell Signaling Technology, USA), and β-actin (1:2000, Abcam).

#### ELISA

ADSCs were treated with a low-concentration diabetic microenvironment (LPS 1 µg/ml, AGE 1 µg/ml, and glucose 2.5 mg/ml) for 24 h. The supernatants of the preconditioned/control groups were collected and the levels of secreted cytokines (IL-6, MCP-1, IL-8, VEGF, IL-10, and TGF-β) were measured using ELISA kits (Neobioscience Technology Co. Ltd, Beijing, China) according to the manufacturer’s instructions.

### Flow cytometric analysis

Once the diabetic microenvironment-preconditioned ADSCs reached 80–90% confluence, the preconditioned and control groups were harvested into centrifuge tubes for incubation with primary antibodies (CD34-PE, CD11a-FITC, CD90-FITC, CD73-PE, CD105-PE, HLA-DR-FITC, F4/80-PE) for 15 min in the dark. A flow cytometer (BD Biosciences) was used to analyse the data. The Annexin V-FITC Apoptosis Detection Kit (BD Biosciences) was used to detect cell apoptosis, as previously described [18].

### Statistical analysis

Data analysis was performed using SPSS 19.0 software and expressed as mean ± standard deviation (SD). Statistical differences (two groups) of normally distributed data or non-normally distributed data were assessed using the unpaired Student *t-*test or Mann–Whitney U test, and differences between three or more groups were evaluated using one-way analysis of variance (ANOVA) with Bonferroni’s multiple comparison test. Statistical significance was set at *P* < 0.05.

## Results

### Secretory ability of ADSCs for anti-inflammation-related factors was enhanced following diabetic microenvironment preconditioning

**Fig. 1:**
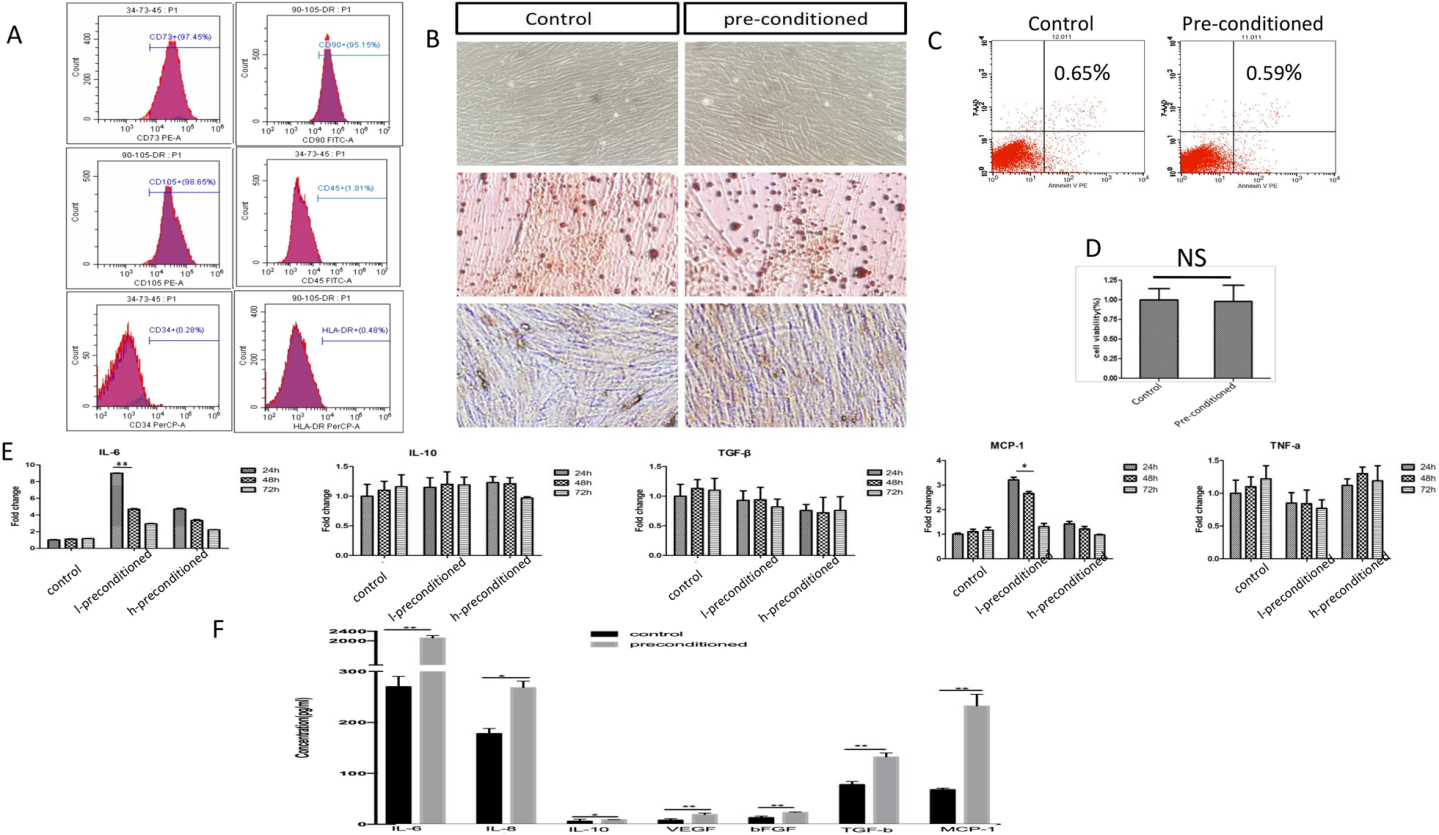
Identification of ADSCs under diabetic microenvironment and the cell secretion effect of ADSCs was enhanced. Immune-phenotype of diabetic microenvironment pre-MSCs by flow cytometry (**A**). Representative photomicrographs of ADSCs and diabetic microenvironment pre-ADSCs and displayed similar spindle shaped morphology (**B**). Annexin V-PE/7-AAD cell apoptosis detected cell necrosis and late apoptosis, and MTT assay is used to detect cell proliferation activity (**C**–**D**). Take different concentrations, stimulate different time, qRT-PCR results of cytokines (**E**). Secreted cytokine of cell supernatant by ELISA test (**F**). Data are presented as the mean ± SEM of three separate experiments. l-precondition: low concentration precondition. h-precondition: high concentration precondition

The characteristics of ADSCs were evaluated under diabetic microenvironment preconditioning (LPS, 1 µg/ml; AGE, 1 µg/ml; and glucose, 2.5 mg/ml; for 24 h). FACS analysis showed that diabetic microenvironment pre-ADSCs were negative for CD34, CD11a, and HLA-DR and positive for CD90, CD73, and CD105. In addition, the pre-ADSCs were found to have a spindle-shaped morphology when observed under an optical microscope (Fig. 1A). Moreover, pre-ADSCs showed the ability to differentiate into adipocytes and osteoblasts (Fig. 1B), and the apoptotic rate of the ADSCs did not increase significantly after preconditioning with the diabetic microenvironment as observed by Annexin V/7-AAD staining (Fig. 1C). These results showed that the diabetic microenvironment pre-ADSCs had similar characteristics to the original ADSCs. Previous studies confirmed that IL-6, monocyte chemoattractant protein-1 (MCP-1), IL-10, TGF-β, TNF-α, and other cytokines secreted by MSCs are closely related to their immunoregulatory effects. Moreover, our previous study showed that IL-6 and MCP-1 are responsible for MSC-induced M2 macrophage polarisation [21]. ADSCs were pretreated with different concentrations of the diabetic microenvironment at different times in our study, and the secretion of IL-6, MCP-1, IL-10, TGF-β, and TNF-α was detected at the transcription and protein levels. According to the results of qRT-PCR, the transcription levels of IL-6 and MCP-1 were highest when ADSCs were pretreated in a low-concentration diabetic microenvironment for 24 h (Fig. 1D). The results of ELISA, which was used to detect the cytokines secreted by cells, were consistent with those of qRT-PCR (Fig. 1E). Therefore, pretreatment with low-concentration diabetic microenvironment for 24 h was selected as the optimal pretreatment condition.

### Compared to ADSCs, co-culture with pre-ADSCs further promoted M2 macrophage polarisation in vitro

**Fig. 2:**
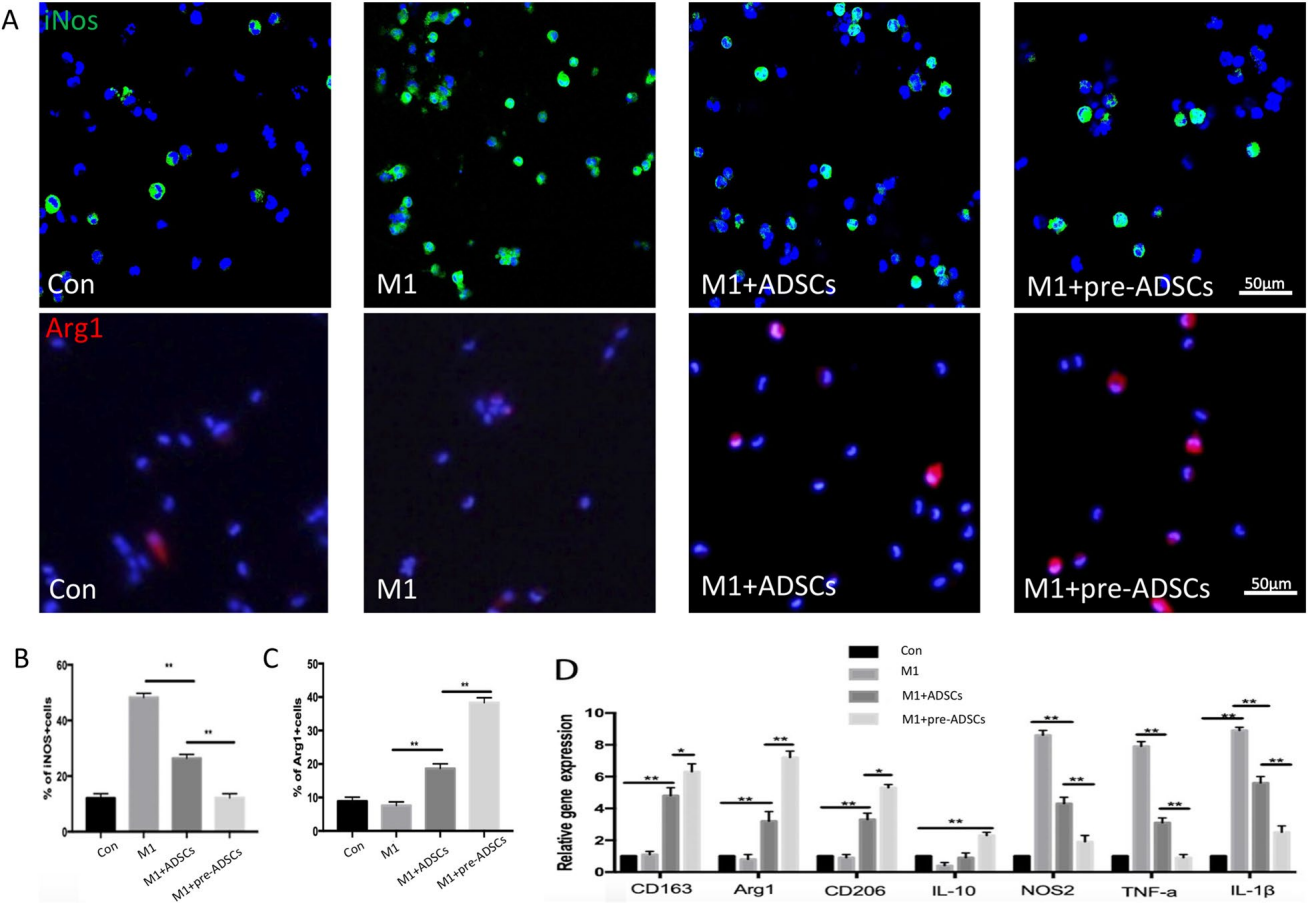
Representative of iNOS-positive or Arg1-positive cells in co-cultured M1 macrophages with control, LPS, ADSCs and pre-ADSCs by optical microscope, immunofluorescence and quantification of iNOS-positive or Arg1-positive cells; bars = 50 μm (**A**–**C**). Quantitative reverse transcriptase polymerase chain reaction analysis of gene expression in the control, LPS, ADSCs and pre-ADSCs groups (**D**); *P < 0.05, **P < 0.01

Next, we sought to determine whether pre-ADSCs would further promote the transformation of macrophages in vitro. Cell-climbing immunofluorescence and PCR were used to detect M1 and M2-related markers (M1: iNOS; M12: Arg1). Macrophages were extracted from peritoneal lavage, and immunofluorescence staining showed that more than 90% of the cells were F4/80 positive (Additional file 2: Fig. S1A). LPS stimulation markedly increased the ratio of iNOS + (M1 macrophage marker) cells to 65.5%, which was reduced to 27.3% by culturing with ADSCs in a transwell system and further reduced to 11.8% following pre-ADSC co-culture (Fig. 2A–C). Additionally, qRT-PCR analysis revealed that compared to the ADSC group, pre-ADSC treatment resulted in higher expression of genes encoding M2 macrophages and anti-inflammatory molecules (Arg1, CD206, CD163, and IL-10) and lower expression of genes encoding M1 macrophages (iNOS, TNF-α, IL-1β) and pro-inflammatory molecules (Fig. 2D). In summary, these results suggest that the ability of ADSCs to promote the polarisation of macrophages from M1 to M2 was significantly enhanced following diabetic microenvironment preconditioning in vitro.

### Compared to ADSCs, multiple pre-ADSC infusions further improved glucose homeostasis by ameliorating insulin resistance and enhancing recovery of pancreatic islets

To examine the effect of pre-ADSCs on glucose homeostasis and long-term T2D complications, we used an HFD diet combined with a one-time injection of low-dose STZ to induce long-term T2D complications in a rat model, as we previously reported (Additional file 2: Fig. S1B). In brief, eight-week-old male SD rats were fed an HFD for 8 weeks. A single dose of STZ (25 mg/kg) was intraperitoneally injected. Rats with more than three random glucose level measurements ≥ 16.7 mmol/L were considered diabetic. Then, the T2D rats were fed a HFD for 24 weeks. The rats were divided into four groups: normal group (Nor group), diabetic untreated group (T2D group), normal ADSC treatment group (ADSCs group), and diabetic microenvironment-preconditioned ADSC treatment group (pre-ADSCs group). Late-stage T2D rats (ADSCs and pre-ADSCs groups) were infused with ADSCs or pre-ADSCs once a week through the tail vein for a total of 6 months.

**Fig. 3:**
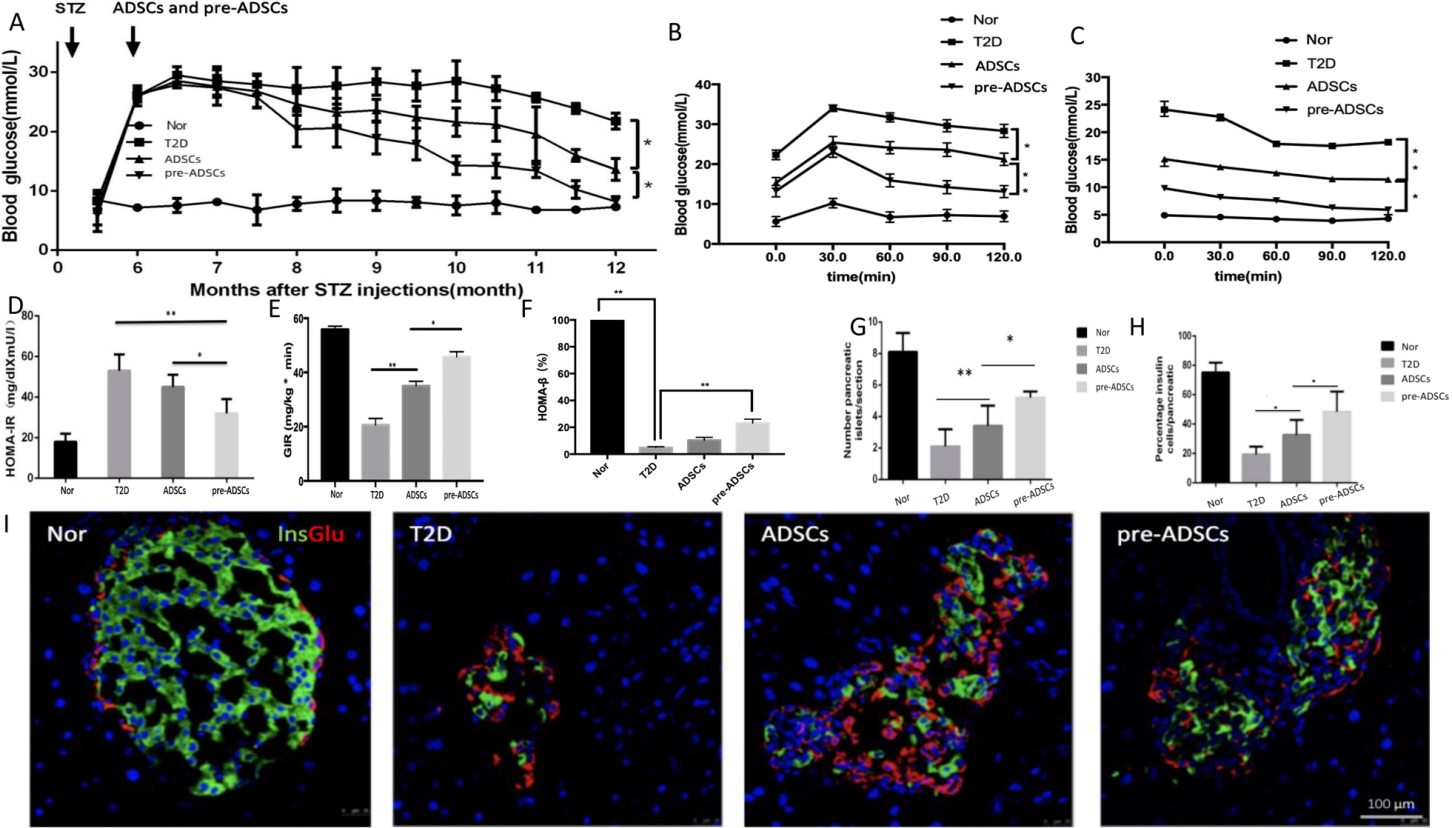
Identification of T2D model and multiple pre-ADSCs infusions improved glucose homeostasis, insulin resistance and islet damage of long-term T2D complication rats. Blood glucose levels were detected consecutively after ADSCs or pre-ADSCs infusions (**A**). Concentration of glucose in the blood of four groups after a IPGTT (**B**) or IPITT (**C**). HOMA-IR of each group (**D**). GIR of hyperinsulinaemic-euglycaemic clamp analysis of each group (**E**). The above findings are all expressed as mean ± standard deviation. The presence and distribution of insulin- (green) and glucagon-producing (red) cells were measured (**I**). Bars = 100 μm (**F**). The β cell mass and percentage of β cells in the pancreatic islets were evaluated (**G**, **H**). *P < 0.05, **P < 0.01

Approximately two months after ADSC infusion, the random blood glucose levels were found to gradually decrease and reached 15.6 ± 1.3 mmol/L at the end of treatment, whereas six-month infusion of pre-ADSCs resulted in nearly normal random blood glucose level (9.8 ± 1.7 mmol/L) (Fig. 3A). Compared to ADSC treatment, IPGTT showed much more improved glucose clearance after pre-ADSC treatment (Fig. 3B). Insulin sensitivity was enhanced following ADSC infusion, as indicated by improvements in IPITT, homeostatic model assessment of insulin resistance (HOMA-IR), and GIR, which were further improved in the pre-ADSC group (Fig. 3C–E). Moreover, compared to the ADSC group, multiple infusions of pre-ADSCs significantly promoted the regeneration of pancreatic β-cells (based on islet numbers) and the proportion of β-cells/islet (Fig. 3F–H). Overall, these results show that compared to ADSCs, multiple pre-ADSC infusions further improved glucose homeostasis by ameliorating insulin resistance and enhancing the recovery of pancreatic islets.

### Compared to ADSCs, multiple pre-ADSC infusions in long-term T2D complication rats further ameliorated T2D-induced kidney diseases

**Fig. 4:**
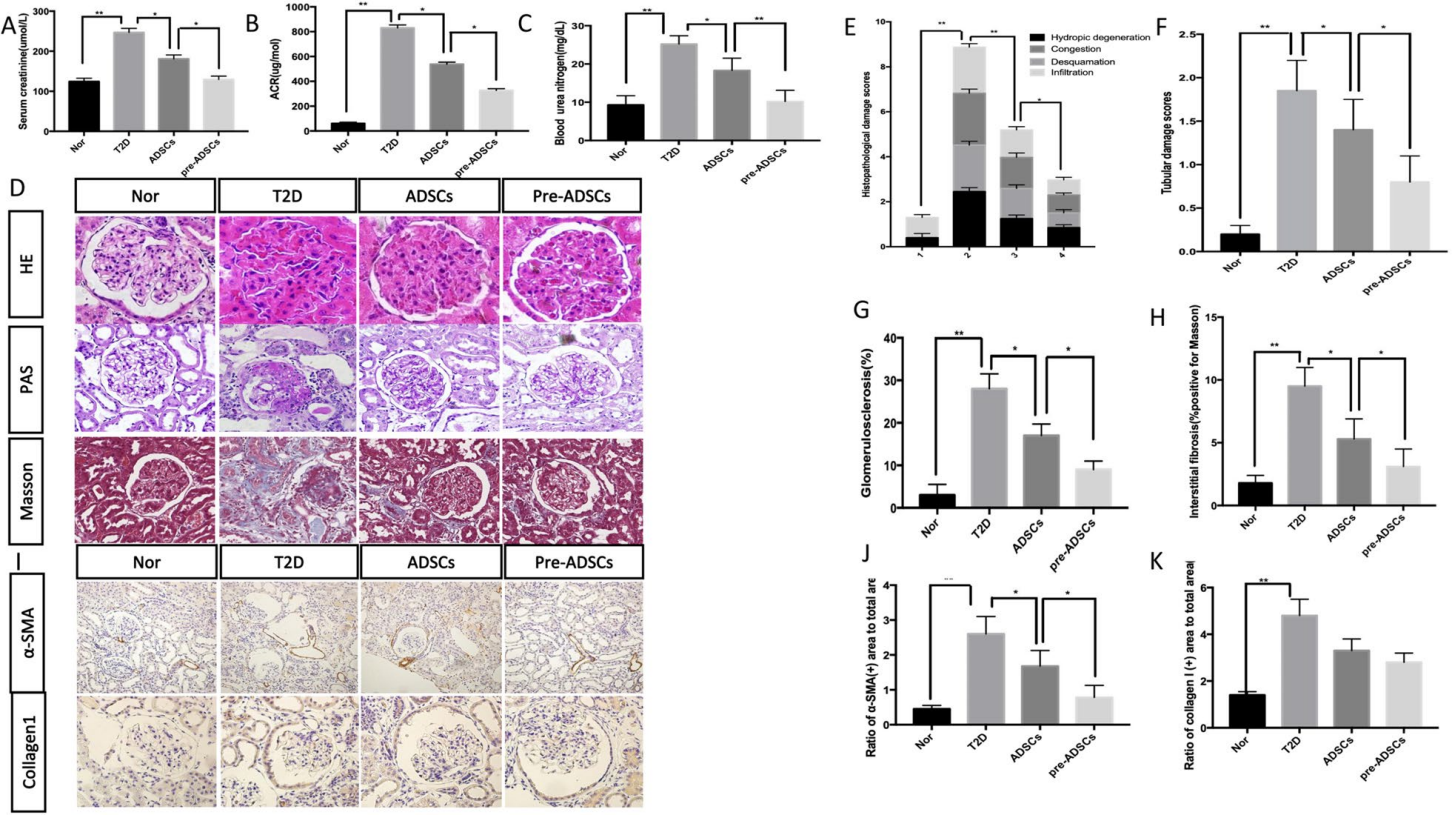
Multiple pre-ADSCs infusions ameliorated long-term T2D induced diseases of kidney. Serum creatinine, blood urea nitrogen, and urine ACR of each group were detected (**A**–**C**). Representative kidney sections by H&E, PAS, and Masson’s trichrome staining; bars = 10, 20 and 20 μm (**D**). Histopathological damage scores and tubular damage score were graded using H&E-stained sections (**E**, **F**). Glomerulosclerotic injury was graded using PAS-stained sections (**G**). Interstitial fibrosis was graded using Masson-stained sections (**H**). Immunohistochemical staining and quantification of α-SMA and col1 areas, bars = 50 and 20 μm (**I**–**K**)

DN is one of the most serious and chronic vascular complications of T2D. Therefore, we evaluated the effects of ADSCs and pre-ADSCs on DN progression. Kidney dysfunction was evident in the T2D rats. Serum creatinine levels were almost doubled (Fig. 4A), the ACR reached 830.2 μg/mmol, which was more than ten-fold higher than that in the normal group (Fig. 4B), and blood urea nitrogen (BUN) reached 25.2 mg/dL (Fig. 4C). All indexes in the pre-ADSC group were further reduced compared to those in the ADSC group. Tubulointerstitial fibrosis, glomerulosclerotic changes, hypertrophy of glomeruli, swelling of tubule cells, and infiltration in T2D rats were obvious with H&E, PAS, and Masson’s trichrome staining. As expected, all kidney damage was significantly attenuated following pre-ADSC treatment compared to that in the ADSC group (Fig. 4D–H). Positive expression of tissue fibrosis markers including collagen type 1 (col1) and alpha smooth muscle actin (α-SMA) was readily observed in the T2D group. The infusion of ADSCs slightly decreased the ratio of α-SMA + area to 64.6% in the kidney, whereas pre-ADSCs substantially reversed this trend with the ratio reducing to 30% (Fig. 4I, J). Treatment with ADSCs also decreased the ratio of collagen I + area, and pre-ADSCs showed a tendency to further reduce this index, although the differences were not statistically significant. Preconditioning clearly improves the protective effects of ADSCs on the kidney.

### Compared to ADSCs, multiple pre-ADSC infusions in long-term T2D complication rats further ameliorated T2D-induced liver diseases

**Fig. 5:**
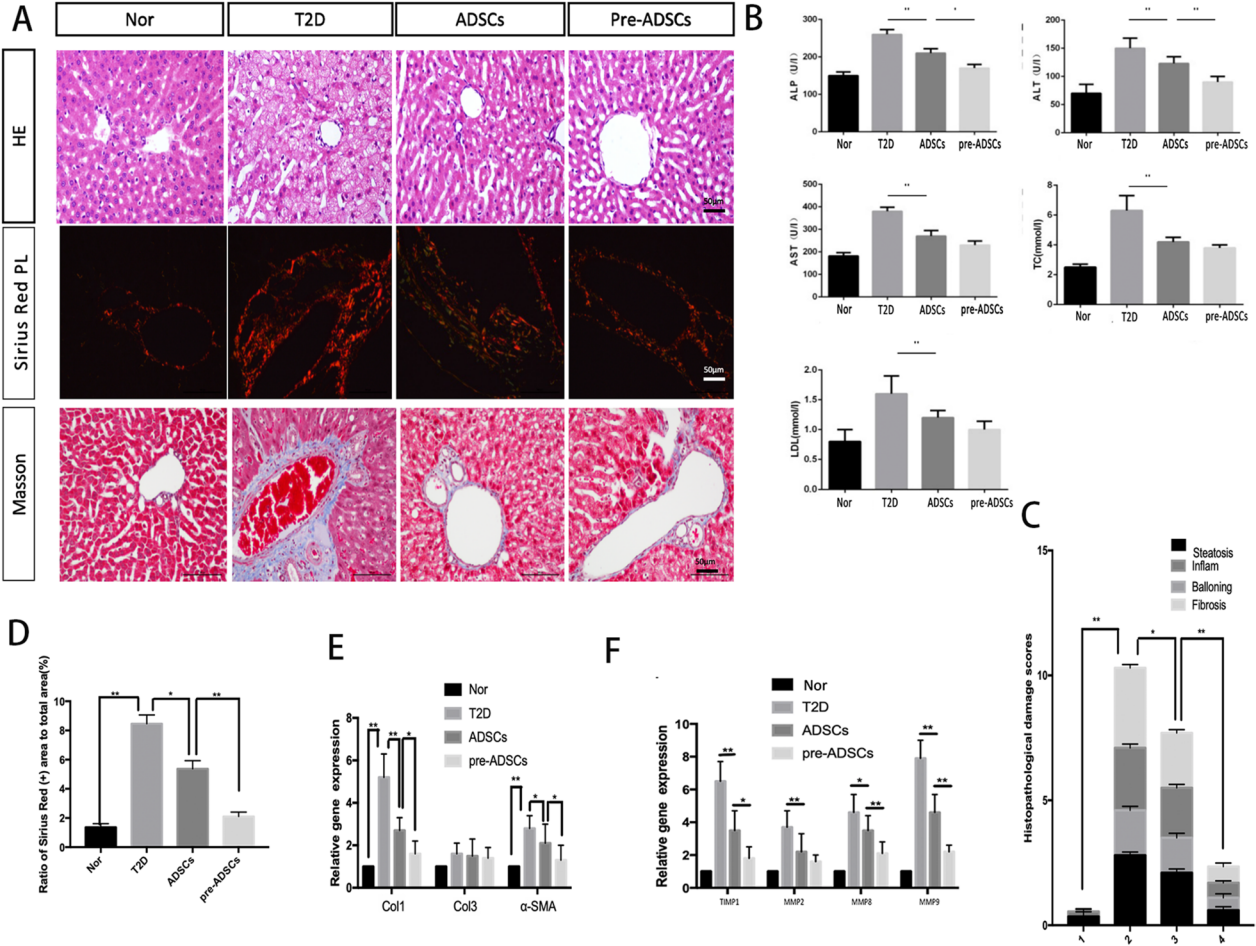
Multiple pre-ADSCs infusions ameliorated long-term T2D induced diseases of liver. Representative liver sections by H&E, PAS, and Masson’s trichrome staining; bars = 50, 50 and 20 μm (**A**). Levels of serum ALP, ALT, AST, LDL-C and TG of each group were evaluated (**B**). Histopathological damage scores were graded using H&E-stained sections (**C**). Ratio of Sirius Red-positive area to total area (**D**). Quantitative reverse transcriptase polymerase chain reaction analysis of gene expression in liver tissue from control, T2D, ADSCs and pre-ADSCs groups (**E**, **F**); results are presented relative to those of control rats, set as 1. N = 6 rats per group; *P < 0.05, **P < 0.01

It is well known that liver fibrosis and fat accumulation contribute to the development of advanced T2D. Therefore, we examined the serum indicators of lipid metabolism and changes in hepatic histopathology. H&E, Sirius Red, and Masson’s trichrome staining revealed that the incidence of liver steatosis, fibrosis features, and inflammation in the pre-ADSC group displayed greater improvement than that in the ADSC group (Fig. 5A, D). The non-alcoholic steatohepatitis (NASH) score, which is used to describe NASH-like features (such as inflammation and steatosis), was decreased by up to 30.52% in the pre-ADSC group compared to the ADSC group (Fig. 5C). The liver partial lipid metabolism disorder levels (including ALP and ALT) in the pre-ADSC group were significantly improved compared to those in the ADSC-treated group (Fig. 5B). Moreover, to further determine whether pre-ADSC treatment could significantly ameliorate liver fibrosis, we detected the expression of profibrogenic genes such as tissue inhibitor of metalloproteinases-1 (TIMP-1), matrix metalloproteinase (MMP-8 and 9), col1, and α-SMA. All these parameters were significantly decreased in the pre-ADSC group compared to those in the other groups. Although the statistical difference was not significant, MMP-2 and col3 levels were slightly decreased in the pre-ADSC group compared to the ADSC group (Fig. 5E, F). These results confirmed the advantages of pre-ADSC infusions in mitigating liver damage caused by long-term T2D compared to ADSCs.

### Compared to ADSCs, multiple pre-ADSC infusions in long-term T2D complication rats further ameliorated T2D-induced cataract and lung diseases

**Fig. 6:**
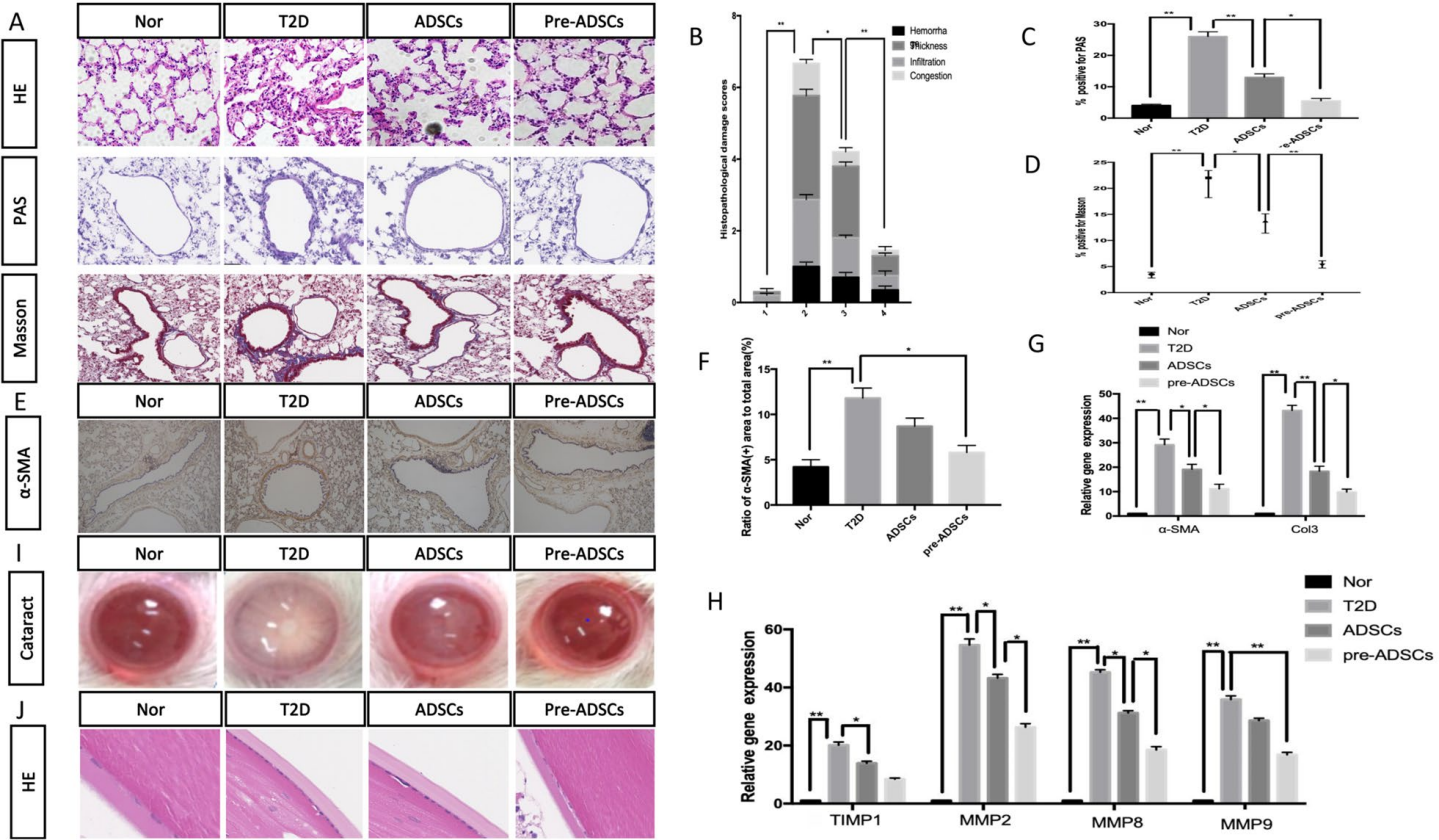
Multiple pre-ADSCs infusions ameliorated long-term T2D induced diseases of lung and eye. Representative lung sections by H&E, PAS, and Masson’s trichrome staining; bars = 20, 10 and 50 μm (**A**). Lung injury scores were evaluated via H&E-stained sections (**B**). Ratio of PAS staining-positive area to whole area (**C**). Ratio of Masson staining-positive area to whole area (**D**). Immunohistochemical staining and quantification of α-SMA area; bars = 100 μm (**E**, **F**). Quantitative reverse transcriptase polymerase chain reaction analysis of gene expression in lung tissue from control, T2D, ADSCs and pre-ADSCs groups; results are presented relative to those of control rats, set as 1. N = 6 rats per group (**G**, **H**); Representative photographs using the camera and representative sections staining with H&E; bars = 50 μm (**I**, **J**), results are presented relative to those of control rats, set as 1. N = 6 rats per group. *P < 0.05, **P < 0.01

A recent study demonstrated obvious structural and physiological abnormalities of the lung in patients with T2D. Therefore, we next evaluated the lungs in the various groups to detect the effect of pre-ADSCs [19]. Compared to the ADSC group, histopathological damage, including infiltration of inflammatory cells, disordered structure of the lung tissue, and alveolar thickness was mitigated effectively in the pre-ADSC group (Fig. 6A), The comprehensive score of lung injury was significantly improved following pre-ADSC treatment (Fig. 6B). In addition, the amelioration of glycogen granules and pulmonary fibrosis were more significant in the pre-ADSC group than in the ADSC group as detected by the percentage of positive PAS-stained areas and Masson-stained areas of collagen (Fig. 6C, D). These findings were consistent with the results of immunohistochemical staining of α-SMA and detection of the expression of related genes including MMP-2, 8 and 9, col3, TIMP-1, and α-SMA (Fig. 6E–H). All these results confirmed that compared to the ADSC group, the total amount of collagen in the alveolar space was reduced following treatment with pre-ADSCs.

Another common complication of diabetes is cataract. After treatment, both ADSC and pre-ADSC groups showed an improvement in the degree of turbidity of the lenses, but the pre-ADSC group showed significantly improved transparent and clearer lenses (Fig. 6I). Furthermore, the anti-cataract effect of pre-ADSC treatment was confirmed by H&E staining (Fig. 6J).

### Compared to ADSCs, multiple pre-ADSC infusions further increased M2 phenotype macrophage polarisation and attenuated tissue inflammation

**Fig. 7:**
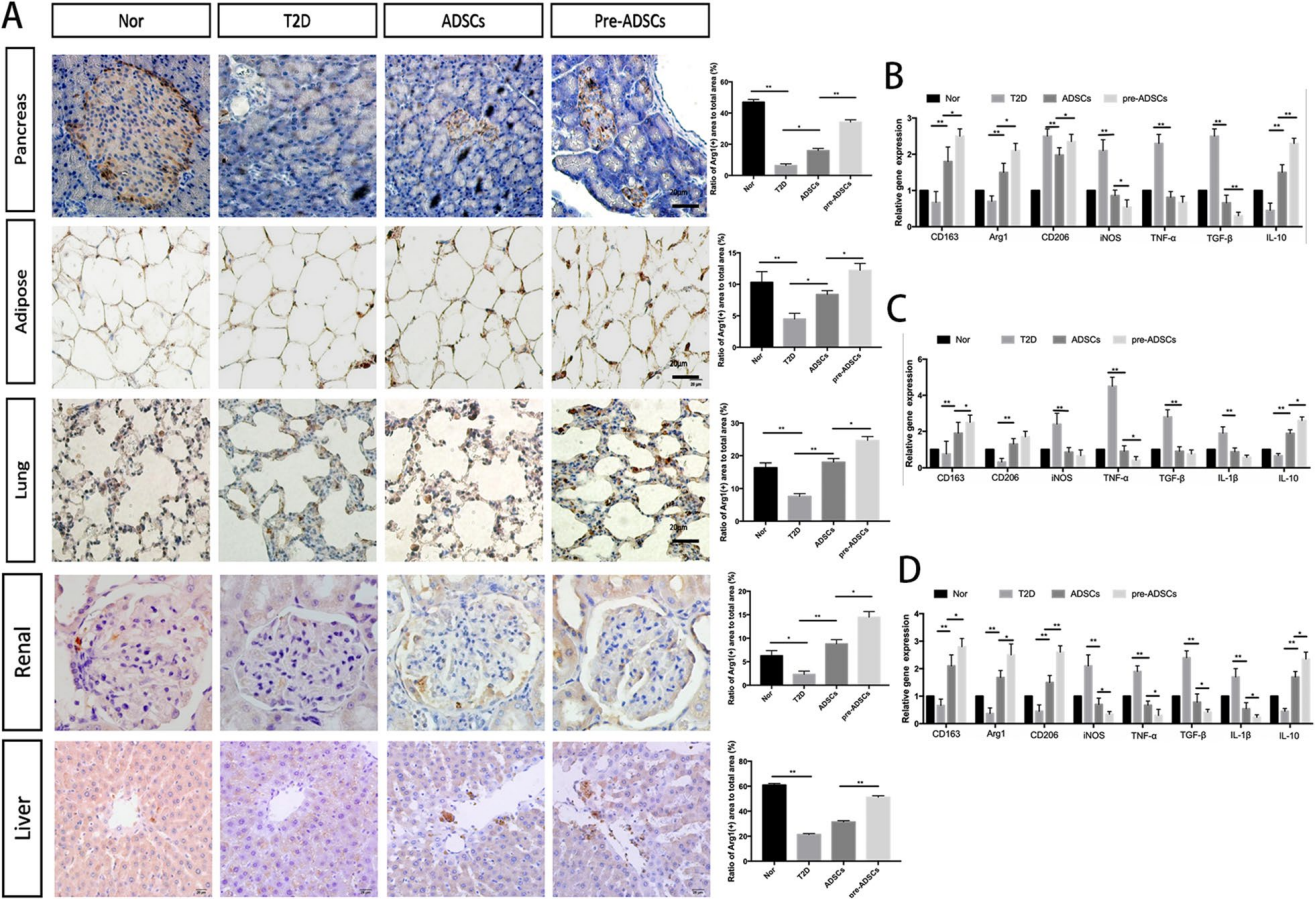
Multiple pre-ADSC infusions changed the phenotypes of macrophages in the target organs in long-term T2D complication rats. Representative Arg1-positive cells in pancreas, adipose, lung, renal, and liver tissue by immunohistochemistry; bars = 20 μm (**A**). Quantitative reverse transcriptase polymerase chain reaction analysis of gene expression in the kidney (**B**), liver (**C**), and lung (**D**) tissue from control, T2D, ADSCs and pre-ADSCs groups; *P < 0.05, **P < 0.01

Next, we analysed whether multiple pre-ADSC infusions further promoted M2 macrophage polarisation and attenuated inflammation in vivo. We analysed the phenotype changes in macrophage in the pancreas, kidney, liver, lung, and adipose tissues of T2D rats. The brown dark-stained cells represent Arg1-positive cells, which represent M2 phenotype macrophages. The proportion of M2 infiltration in each tissue from the pre-ADSC treatment group was significantly higher than that from the T2D and ADSC groups (Fig. 7A, B). Likewise, in the pre-ADSC group, the genes encoding CD163, CD206, and Arg1 (M2 macrophage markers) were highly expressed, and the expression of iNOS (M1 macrophage marker) was lower (Fig. 7C–E). Additionally, qRT-PCR analysis of tissues showed that the expression of genes related to anti-inflammation (IL-10) was enhanced whereas those related to fibrosis and inflammation (TGF-β, TNF-α, IL-1β) was decreased in the pre-ADSC group.

## Discussion

Several lines of evidence have shown that MSCs have the potential to exert therapeutic effects against diabetes-related complications [22–24]. However, few studies have reported ways to enhance the therapeutic effects of MSCs in long-term T2D complications. The present study showed that pre-ADSCs treated with LPS, AGE, and high glucose had significantly greater advantages in the treatment of the final stage of T2D than MSCs, especially in regulating blood glucose (BG) homeostasis, promoting islet regeneration, and improving T2D-related complications (lung fibrosis, CKD, hepatic steatosis and fibrosis, and cataract).

Macrophages show a range of phenotypes between two extremes, the M1 phenotype tends to be pro-inflammatory and the M2 phenotype tends to be anti-inflammatory. Our previous studies have confirmed that MSCs promote the conversion of macrophages from the M1 phenotype to the M2 phenotype by secreting cytokines such as IL-6 and MCP-1 [21, 25] in islets and adipose tissue, and the conversion of macrophage phenotype results in reduced secretion of M1 inflammatory factors. In the present study, pretreatment with inflammatory factor, high glucose, and AGE in vitro increased the secretion of MCP-1 and IL-6. Moreover, pre-ADSCs promoted M2 macrophage polarisation in vitro compared to ADSCs. When injected into long-term T2D rats, pre-ADSCs further increased the number of M2 macrophages and attenuated inflammation in multiple tissues. Therefore, we concluded that diabetic environmental conditions enhanced the therapeutic effects of ADSCs on glucose homeostasis, islet regeneration, and long-term T2D complications, at least partially, via stimulation of ADSCs to increase the expression of cytokines (IL-6 and MCP-1) which regulate macrophage polarisation. Further confirmation of this conclusion would require testing whether blocking the expression of IL-6 and MCP-1 in pre-ADSCs inhibits M2 macrophage polarisation and abrogates the therapeutic effects, and needs to be addressed in future experiments.

Why does diabetic microenvironmental pretreatment enhance the therapeutic effects of ADSCs in BG homeostasis and diabetic complications in late-stage T2D? First, according to recent data, the secretion profile of MSCs may be influenced by the environment. For instance, Krampera et al. first found that treatment with IFN-γ results in the expression of IDO and increases the expression of PGE2 and HGF in MSCs [26]. Another study showed that the expression of proteins such as PD-L1, IDO, and IL-6 is dramatically upregulated under inflammatory conditions [27]. Consistently, our study confirmed that ADSCs secrete increased IL-6 and MCP-1 under conditions of diabetic microenvironmental pretreatment. A series of experiments have confirmed that MSCs have short-term memory capabilities that are similar to those of immune cells. Once MSCs are stimulated with an appropriate concentration of LPS or TNF-α, the expression of IL-6 and MCP-1 increases, then following the removal of the stimulating factors for a period, IL-6 and MCP-1 can increase rapidly in a short period of time when a similar stimulation is given again. This may be because the expression of receptors on the surface of MSCs changes. A study confirmed that LPS activates TLR4 on the MSC surface, thereby increasing the expression of IL-6, IL-8, CCL2, and CXCL1 [28]. Based on this theory, pretreatment with inflammation, high glucose, and AGE in vitro may alter the expression of certain receptors of MSCs, leading to increased secretion of MCP-1 and IL-6. When pre-ADSCs are infused into T2D rats, exposure of the cells to an environment similar to the in vitro culture environment may cause them to release a large amount of cytokines, such as MCP-1 and IL-6, thereby significantly enhancing the therapeutic ability of the MSCs.

Previous reports have confirmed that the therapeutic effect of a single infusion lasted for a limited duration in the early onset T2D/diabetic complication model. However, multiple infusions can achieve long-term amelioration of T2D/diabetic complications [15, 29, 30]. In the present study, we demonstrated that injection of pre-ADSCs once a week for 24 weeks restored BG to normal levels and markedly reversed DN in the late-stage T2D/diabetic complications model. However, the question of the potential side effects, such as increased risk of tumours, and myocardial and cerebral infarction, of continuous multiple infusions remains a concern. In our study, no tumours, myocardial infarction, or cerebral infarction were observed after the infusion of ADSCs and pre-ADSCs. Notably, the above results suggest that it is critical to determine the optimal number of infusions through randomised controlled trials in clinical practice.

## Conclusion

In conclusion, MSCs pretreated in the diabetic microenvironment (LPS, high glucose, and AGE) had better influences for maintaining BG homeostasis, reducing tissue IR, promoting the ability of islet repair and ameliorating related complications compared with the untreated MSCs. The pre-MSCs promoted the transformation of macrophages from M1 to M2 through secreting effects. These findings provide novel insights that the use of pretreatment methods can amplify the secretion effect of MSCs, and apply cell culture products to clinics, which can achieve safe and effective treatment.

## Supplementary Information

**Additional file 1: Table 1.** The primers used in quantitative real-time reverse transcriptase polymerase chain reaction (qRT-PCR)

**Additional file 2: Fig. S1.** Identification of peritoneal macrophages and Illustration for the study design. Representative of F4/80-positive cells of peritoneal macrophages by immunofluorescence staining bars = 50 μm (A); To produce the long-term T2DM complication rodent model, 8-week-old male SD rats were fed a HFD for 8 weeks, followed by an STZ injection at a single dose of 25 mg/kg. HFD feeding and hyperglycaemia were maintained in the newly diabetic rats for 26 weeks. Then, the long-term T2DM complication rats were randomly treated with one of the following interventions: infusions of 3 × 106 pre-ADSCs or ADSCs suspended in 0.5 ml of PBS through the tail vein once a week for 26 weeks (referred to as the pre-ADSCs and ADSCs-treated groups, N = 6 and 6) or infusions of 0.5 ml PBS alone once a week for 26 weeks (referred to as the T2DM group, N = 6). Normal rats of the same age that fed NCD were used as the control (referred to as the control group, N = 6). On week 52 (after 26 times of ADSC treatment), the therapeutic effects of ADSCs on T2DM complications were assessed (B).

## Data Availability

The datasets used and/or analysed during the current study are available.

## Abbreviations

MSCs: Mesenchymal stem cells
T2D: Type 2 diabetes mellitus
DN: Diabetic nephropathy
STZ: Streptozotocin
HFD: High-fat diet
ADSCs: Adipose- derived mesenchymal stem cells
CKD: Chronic kidney disease
NASH: Nonalcoholic steatohepatitis
SD: Sprague–Dawley
FBS: Foetal bovine serum
PBS: Phosphate buffer solution
NCD: Normal chow diet
IPGTTs: Intraperitoneal glucose tolerance tests
IPITT: Insulin tolerance tests
FBI: Fasting blood insulin
FBG: Fasting blood glucose
TC: Total cholesterol
LDL-C: Low-density lipoprotein cholesterol
TG: Triglyceride
ALT: Alanine aminotransferase
AST: Aspartate aminotransferase
BUN: Blood urea nitrogen
ACR: Albumin to creatinine ratio
HOMA-IR: Homeostatic model assessment index of insulin resistance
HOMA-β: Homeostatic model assessment index of β cells
CM-Dil: Chloromethyl- benzamidodialkylcarbocyanine
α-SMA: α-Smooth muscle actin
TIMP-1: Tissue inhibitor of metalloproteinases-1
MMP: Matrix metalloproteinase
col1: Collagen type I
col3: Collagen type III
AGE: Advanced glycation end-product
LPS: Lipopolysaccharides
