# Supplementary material for: Diabetic microenvironment preconditioning of adipose tissue-derived mesenchymal stem cells enhances their anti-diabetic, anti-long-term complications, and anti-inflammatory effects in type 2 diabetic rats
Source: Stem Cell Res Ther. 2022 Aug 19;13:422. doi: 10.1186/s13287-022-03114-5 (PMC9389728; doi:10.1186/s13287-022-03114-5)
Supplement: Supplementary file 1 — Additional file 1: Table 1. The primers used in quantitative real-time reverse transcriptase polymerase chain reaction (qRT-PCR) [file 13287_2022_3114_MOESM1_ESM.docx]

Supplementary Table 1: The primers used in quantitative real-time reverse transcriptase polymerase chain reaction (qRT-PCR)

| Genes | Primer pairs |
| --- | --- |
| Nos2 | For: CCAACCTGCAGGTCTTCGATG |
|  | Rev: GTCGATGCACAACTGGGTGAAC |
| IL10 | For: ATGGCCCAGAAATCAAGGAGC |
|  | Rev: GAAGATGTCAAACTCATTCATGGCC |
| CD163 | For: TGTAGTTCATCATCTTCGGTCC |
|  | Rev: CACCTACCAAGCGGAGTTGAC |
| TNFα | For: TCCGCAGATACCTGGAACTC |
|  | Rev: CTCAGATCCTCCCCATTCAA |
| Arg1 | For: CCAAGCCAAAGCCCATAGAG |
|  | Rev: TCCTCGAGGCTGTCCCTTAG |
| IL-1β | For: TACCTATGTCTTGCCCGTGGAG |
|  | Rev: GAAGATGTCAAACTCATTCATGGCC |
| β- actin | For: GAGAGGGAAATCGTGCGTGAC |
|  | Rev: CATCTGCTGGAAGGTGGACA |
| IL-6 | For: CCGGAGAGGAGACTTCACAG |
|  | Rev: TGACAGTGCATCATCGCTGTTC |
| TGFβ | For: ATACGCCTGAGTGGCTGTCT |
|  | Rev: TTGGGACTGATCCCATTGAT |
| VEGF | For: GCCCATGAAGTGGTGAAGTT |
|  | Rev: ACTCCAGGGCTTCATCATTG |
| MCP-1 | For: CGTGCTGTCTCAGCCAGAT |
|  | Rev: GGATCATCTTGCCAGTGAATG |
| CD206 | For: ACTGCGTGGTGATGAAAGG |
|  | Rev: TAACCCAGTGGTTGCTCACA |
