# Supplementary figures and images for: Diabetic microenvironment preconditioning of adipose tissue-derived mesenchymal stem cells enhances their anti-diabetic, anti-long-term complications, and anti-inflammatory effects in type 2 diabetic rats
Source: Stem Cell Res Ther. 2022 Aug 19;13:422. doi: 10.1186/s13287-022-03114-5 (PMC9389728; doi:10.1186/s13287-022-03114-5)

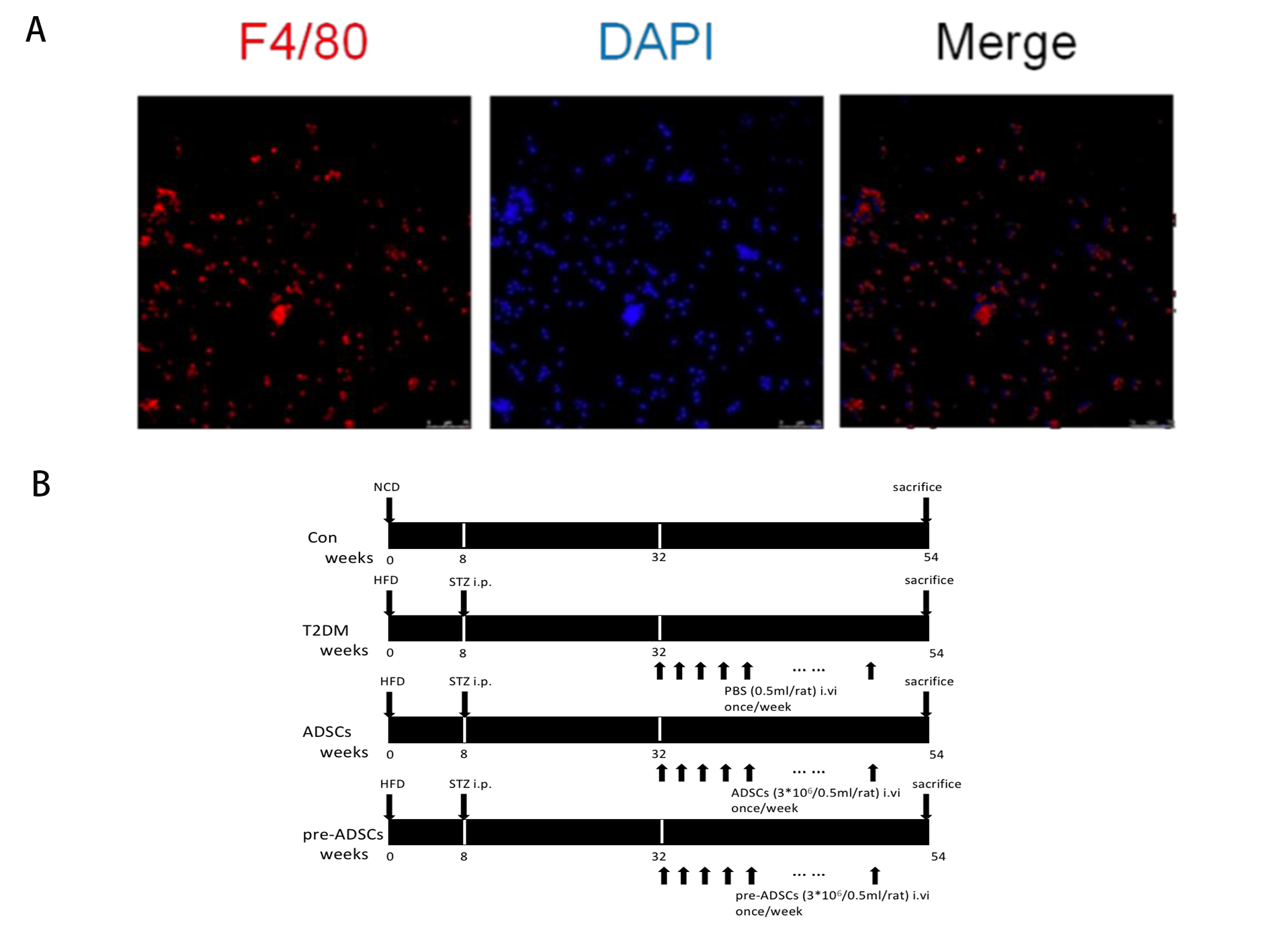

Supplement: Supplementary file 2 — Additional file 2: Fig. S1. Identification of peritoneal macrophages and Illustration for the study design. Representative of F4/80-positive cells of peritoneal macrophages by immunofluorescence staining bars = 50 μm (A); To produce the long-term T2DM complication rodent model, 8-week-old male SD rats were fed a HFD for 8 weeks, followed by an STZ injection at a single dose of 25 mg/kg. HFD feeding and hyperglycaemia were maintained in the newly diabetic rats for 26 weeks. Then, the long-term T2DM complication rats were randomly treated with one of the following interventions: infusions of 3 × 10^6^ pre-ADSCs or ADSCs suspended in 0.5 ml of PBS through the tail vein once a week for 26 weeks (referred to as the pre-ADSCs and ADSCs-treated groups, N = 6 and 6) or infusions of 0.5 ml PBS alone once a week for 26 weeks (referred to as the T2DM group, N = 6). Normal rats of the same age that fed NCD were used as the control (referred to as the control group, N = 6). On week 52 (after 26 times of ADSC treatment), the therapeutic effects of ADSCs on T2DM complications were assessed (B). [file 13287_2022_3114_MOESM2_ESM.tif]
